# Vertical Movements and Patterns in Diving Behavior of Whale Sharks as Revealed by Pop-Up Satellite Tags in the Eastern Gulf of Mexico

**DOI:** 10.1371/journal.pone.0142156

**Published:** 2015-11-18

**Authors:** John P. Tyminski, Rafael de la Parra-Venegas, Jaime González Cano, Robert E. Hueter

**Affiliations:** 1 Center for Shark Research, Mote Marine Laboratory, Sarasota, Florida, United States of America; 2 Ch'ooj Ajauil AC, Cancún, Quintana Roo, México; 3 Proyecto Dominó, Comisión Nacional de Áreas Naturales Protegidas, Cancún, Quintana Roo, México; Griffith University, AUSTRALIA

## Abstract

The whale shark (*Rhincodon typus*) is a wide-ranging, filter-feeding species typically observed at or near the surface. This shark’s sub-surface habits and behaviors have only begun to be revealed in recent years through the use of archival and satellite tagging technology. We attached pop-up satellite archival transmitting tags to 35 whale sharks in the southeastern Gulf of Mexico off the Yucatan Peninsula from 2003–2012 and three tags to whale sharks in the northeastern Gulf off Florida in 2010, to examine these sharks’ long-term movement patterns and gain insight into the underlying factors influencing their vertical habitat selection. Archived data were received from 31 tags deployed on sharks of both sexes with total lengths of 5.5–9 m. Nine of these tags were physically recovered facilitating a detailed long-term view into the sharks’ vertical movements. Whale sharks feeding inshore on fish eggs off the northeast Yucatan Peninsula demonstrated reverse diel vertical migration, with extended periods of surface swimming beginning at sunrise followed by an abrupt change in the mid-afternoon to regular vertical oscillations, a pattern that continued overnight. When in oceanic waters, sharks spent about 95% of their time within epipelagic depths (<200 m) but regularly undertook very deep (“extreme”) dives (>500 m) that largely occurred during daytime or twilight hours (max. depth recorded 1,928 m), had V-shaped depth-time profiles, and comprised more rapid descents (0.68 m sec^-1^) than ascents (0.50 m sec^-1^). Nearly half of these extreme dives had descent profiles with brief but conspicuous changes in vertical direction at a mean depth of 475 m. We hypothesize these stutter steps represent foraging events within the deep scattering layer, however, the extreme dives may have additional functions. Overall, our results demonstrate complex and dynamic patterns of habitat utilization for *R*. *typus* that appear to be in response to changing biotic and abiotic conditions influencing the distribution and abundance of their prey.

## Introduction

The whale shark (*Rhincodon typus* Smith, 1828) is a wide-ranging filter-feeding species that occurs throughout tropical, subtropical, and warm temperate oceans [1,2]. Although directed fisheries for whale sharks have largely been banned or limited in recent years, *R*. *typus* is still landed in certain parts of its range [3–5]. The species is protected in a number of countries due to its high value in international trade, K-selected life history, highly migratory and docile nature, and low abundance [6]. *R*. *typus* is listed as ‘Vulnerable’ in the IUCN Red List of Threatened Species [7] and is one of eight shark species currently listed on Appendix II of the Convention on International Trade in Endangered Species of Wild Fauna and Flora (CITES). Effective conservation strategies for highly migratory species such as the whale shark require some understanding of the biotic and abiotic factors driving the animals’ movements. Although the use of satellite tag technology has helped advance our knowledge of whale shark horizontal movements in the Atlantic Ocean [8] and other parts of its range [9,10], the sub-surface habits and movement behaviors of this species have not been well studied, particularly when the sharks are occupying offshore pelagic habitats.

Some studies have demonstrated these planktivores spend the majority of their time in the upper 10 m of the water column [11–13] but also undertake frequent vertical oscillations within the epipelagic zone [14,15]. Evidence of diel and crepuscular patterns in the vertical movements of *R*. *typus* has shown depths during daytime generally greater than nighttime and swimming patterns often abruptly changing at dawn or dusk [15]. Repetitive epipelagic dives can be punctuated by mesopelagic and bathypelagic dives when in oceanic waters [12,15,16]. An immature female satellite-tagged in coastal waters off Mozambique demonstrated regular dives in oceanic waters to at least 1,286 m (the maximum the tag could measure) but spent little time at the maximum depth [17]. These authors suggested these deep dives were related to foraging behavior when crossing comparatively less productive oceanic surface waters. The use of multi-sensor data-loggers has indicated that whale sharks at Ningaloo Reef, Western Australia glide during their descents and may, at times, harness their negative buoyancy to minimize the horizontal cost of transport [18]. Collectively these studies suggest that whale shark diving behavior is complex and can change in relation to time, location, and bathymetry [19]. However, a clear understanding of the ecological and/or physiological importance of these patterns remains elusive.

The goal of our study was to characterize the vertical habitat by depth and temperature utilized by whale sharks of different sizes and sexes in the Gulf of Mexico (GoM). Between mid-April and September each year, whale sharks aggregate in the continental shelf waters off the northeast corner of Mexico’s Yucatan Peninsula, where the northwestern Caribbean Sea meets the southeastern GoM [8,20]. This aggregation site, which is primarily a feeding area for these planktivorous sharks, provides an ideal area to study whale sharks using satellite tag technology as both sexes at various stages of maturity are reliably found in large numbers by the middle of the summer season [20,21]. In recent years, studies utilizing pop-up satellite archival transmitting tags (PSATs) on sharks have revealed detailed movement patterns and habitat utilization that were previously unobservable [22–24]. This type of archival tag can deliver high resolution data and contribute to our knowledge of the complex temporal and behavioral dynamics of these animals [25]. By analyzing the fine-scale depth data from recovered PSATs, we aimed to identify periodicities and patterns in the dive records and gain insight into the underlying factors that influence habitat selection and behavior of the whale shark. Lastly, we sought to examine the time/depth profiles and dive rates of whale shark dives deeper than 500 m, to better understand the function of these extreme dives and provide insight into the ecology and physiology of this deep-diving elasmobranch.

## Methodology

Research for this publication was carried out with prior permission from the Mexican federal government agency CONANP and was reviewed and approved by the Institutional Animal Care and Use Committee at Mote Marine Laboratory.

### Study Area and Tagging Procedures

**Fig 1:**
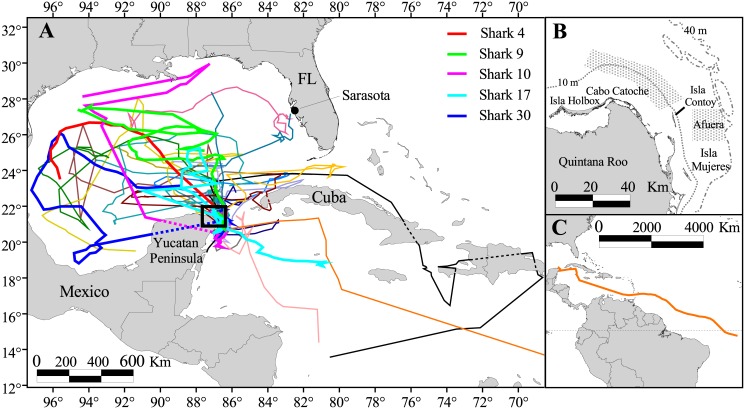
Study sites for the tagging of *Rhincodon typus* in the Eastern Gulf of Mexico. (A) The most probable tracks of whale sharks satellite-tagged off the northeast Yucatan Peninsula (n = 22) and Florida’s west coast (n = 2). The long distance tracks from recovered tags are shown with a thicker trace and identified in the legend. (B) The two main aggregation sites off the Yucatan Peninsula, north of Cabo Catoche and east of Isla Contoy (Afuera). (C) The most probable track of Shark 15 (Rio Lady). Portions of tracks that cross land due to temporal gaps in the data are shown with a broken line. Reprinted from [8] under a CC BY license, with permission from [Robert E. Hueter], original copyright [2013].

The GoM is a semi-enclosed sea approximately 1,600 km from east to west and 900 km from north to south and is bounded by the United States, Mexico, and Cuba (Fig 1A). Maximum known depth in the GoM is 4,384 m (Sigsbee Deep) with an average depth of 1,615 m [26]. Between 2003 and 2012, whale sharks were located each summer (July-Sept) off Quintana Roo, Mexico on the Yucatan Peninsula in one of two primary sites: one north of Cabo Catoche in the southeastern GoM and the other east of Isla Contoy (hereafter referred to as the Afuera meaning the “outside” aggregation) in the Caribbean Sea (Fig 1B). The Cabo Catoche site is characterized by relatively turbid, shallow green water (6–20 m) with dense patches of crustacean zooplankton [21] while the Afuera is a deeper area (20–40 m) of clear blue water with dense homogenous patches of fish eggs (primarily from the little tunny, *Euthynnus alletteratus*) during the summer [20]. In addition to the field work in Mexico, related studies were conducted in the northeastern GoM off Sarasota, Florida during May and June of 2010. From all areas combined a total of 38 whale sharks were tagged with PSAT tags (1 PAT2, 6 PAT4, 31 Mk10-PAT; Wildlife Computers, Redmond, WA, USA). The Quintana Roo study area, equipment, and tagging procedures are described in detail by Hueter et al. (2013) [8]. Briefly, the PSATs archived ambient water temperature, pressure, and light level measurements while attached to the animal for a user-determined duration (30–200 days). The parameters were sampled at varying intervals (from 3 to 60 s) with tags programmed to store depth and temperature in bins of either 4, 6, or 8 h of the following ranges: depth 0–3, 3–6, 6–10, 10–20, 20–50, 50–100, 100–200, 200–300, 300–400, 400–500, 500–750, 750–1000, and >1000 m; and temperature 0–3, 3–6, 6–9, 9–12, 12–15, 15–18, 18–21, 21–24, 24–27, 27–30, 30–33, and >33°C. This provided time-at-depth and time-at-temperature histograms as well as profiles of water temperature at depth (PAT2 and PAT4 tags accommodated 12 bins and hence were not always directly comparable to Mk10 summary data). The tag tethers were equipped with pressure-activated guillotines to sever the tether if exposed to extreme depth (RD1500 up to 2006, changed to RD1800 after 2006; Wildlife Computers). The number associated with the RD device refers to the activation depth (m). At the Quintana Roo tagging locations, water depth to bottom ranged 8–43 m and sea surface temperature (SST) and salinity varied 24.0–30.0°C and 33.5–35.8 ppt, respectively. At the Florida sites, depth to bottom was 16–27 m and SST and salinity ranged 27.8–29.1°C and 33.3–34.6 ppt, respectively. Each shark’s total length was estimated to the nearest 0.5 m and sex and maturity status were recorded using methods described by Hueter et al. (2013) [8]. Once detached and at the sea surface, tags transmitted summaries of archived data through the Argos satellite system. Physically recovered PSATs enabled the direct retrieval of their full archived data sets.

### PSAT Analysis

Summary data received through the Argos satellite system and directly downloaded data from recovered tags were both processed using the tag manufacturer’s proprietary software (WC-AMP v. 1.2.15 and WC-GPE v. 1.02.005; Wildlife Computers). Most probable tracks (MPTs; Fig 1) were estimated using the methods detailed by Hueter et al. (2013) [8]. Briefly, daily light-based geolocations were estimated using GPE software. The raw tracks were smoothed with the state-space unscented Kalman filter which incorporated tag-measured SSTs to predict the MPT. Lastly, we applied a secondary bathymetric correction that constrained estimated locations based on daily maximum depths that the shark achieved. Comparisons from a double-tagging experiment with a secondary satellite tag calculating the transmitter’s position by Doppler shift indicated that the approach resulted in reliable and statistically sound tracks [8].

### Fast Fourier Transform Analysis

A fast Fourier transform (FFT) analysis was performed to identify periodicities in the high resolution data sets from physically recovered PSATs. The FFT decomposes complex time-series data into a function of sine and cosine terms of different frequencies [27] and is appropriate for analyzing the fine-scale data that can be attained from archival tags [28]. A periodogram was utilized to visualize the frequencies and magnitude of any periodic components in the data as represented by spectral peaks that stood above the background noise. The FFT with a ‘hamming’ window function [28,29] was applied to the depth data that were first corrected for pressure-sensor drift. All FFT analyses were performed with Igor Pro software (vers. 6.1.1.0).

### Other Calculations and Statistical Analyses

Data from recovered tags were visually inspected using Wildlife Computers’ Instrument Helper (vers. 3.0) and exported for analysis and graph construction using Igor Pro (vers. 6.1.1.0) and SigmaPlot (vers. 10.0). To evaluate the mean time spent in the tag’s user-determined depth and temperature bins, we pooled the data from tags (both recovered and those reporting via satellite) with identically structured bins and with deployment durations of 30 days or more. Descent and ascent velocities (m s^-1^) were calculated over the 3, 30, or 60 second intervals recorded by the PSAT tag. Tag-recorded light measurements were the primary means of distinguishing day, night, and twilight records. If a shark was at depth during periods of light transition, we consulted known sunrise/sunset times given the sharks’ MPT using an online calculator (http://sunrisesunsetmap.com). To detect day vs night differences in mean depth for individual sharks, we performed two-sample t-tests. To evaluate differences in percent time-at-depth/temperature between sexes, sizes, and day vs night, we performed two-sample Kolmogorov-Smirnov (K-S) tests. These statistical analyses were performed using the Stats package for R [30].

## Results

**Fig 2:**
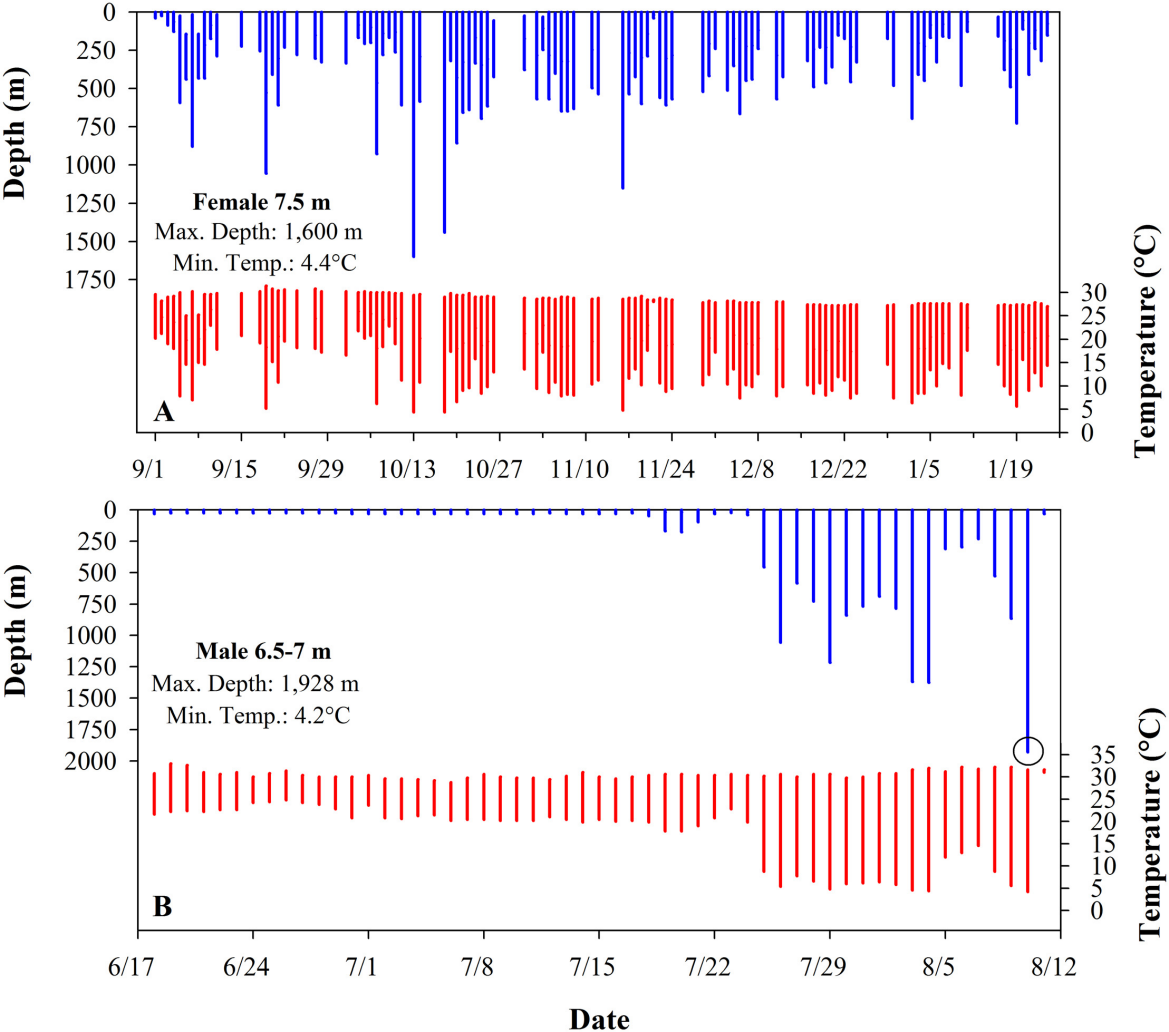
The daily range in depth (blue) and temperature (red) from satellite summary data for two whale sharks (*Rhincodon typus*). (A) Shark 15 (Rio Lady) traveled farther than any other shark in the study (7,772 km). (B) Shark 37 demonstrated the deepest dive of the study (1,928 m) as indicated by an open circle.

We received data from 31 of the 38 deployed PSAT tags (19 females, 12 males) at large from 2 to 190 days (Table 1). Nine of these tags were physically recovered yielding >10 million archived records of depth, temperature, and light. One of the recovered tags (Shark 17) ran out of archive memory such that only 82 days of the 97-day track were recovered in high resolution. Of the two Florida-tagged sharks with substantial movements, one moved to the north in the northeastern GoM (Shark 37) while the other took a southeasterly track that brought it to the northeast Yucatan Peninsula before moving back north into the upper GoM (Shark 36). Mexico-tagged sharks remained in the vicinity of the tagging area until the fall (late August through mid-October) and then dispersed throughout the GoM, through the Straits of Florida, and into the Caribbean Sea (Table 1 and Fig 1A). Shark 15 (nicknamed Rio Lady) traveled to the South Atlantic Ocean, a route of nearly 7,800 km, and dove to at least 1,600 m (Figs 1C and 2A). Sharks regularly moved from surface waters to depths in excess of 1,000 m and experienced temperatures ranging 4.2–33.0°C (Table 1 and Fig 2). The maximum depth recorded for any whale shark in our study was 1,928 m by a 6.5–7 m male (Shark 37) on August 10, 2010 (Fig 2B). Based on the MPT (Fig 1A), this dive occurred in the north-central GoM, approximately 379 km southwest of the Mississippi River delta, where depth to the bottom was about 2,115 m. Extreme depth triggered the activation of the tag tether’s depth failsafe for Shark 37 (RD1800) and Shark 9 (RD1500) and prematurely released these tags to avoid damage due to pressure. It is reasonable to assume that whale sharks in the GoM occasionally dive to depths in excess of 2,000 m.

**Table 1:** Summary of results from PSATs deployed on whale sharks (*Rhincodon typus*) off the northeastern Yucatan Peninsula, Mexico and west Florida 2003–2012 (n = 31).

Shark No.	Sex	Est. TL (m)	Tagging Date	Days of Data	Max. Depth (m)	Min. Temp. (°C)	Max. Temp. (°C)	Generalized Movement†
4*	M	6	8/31/05	31	954	4.5	31.3	GoM
5	M	5.5	2/05/09	30	304	11.4	30.2	Carib.
6*	M	5.5	7/20/06	2	11	20.3	27.3	Cabo Site
7*	F	7	7/21/06	18	17	19.6	26.9	Cabo Site
9*	F	8.5	7/22/06	190	1,504	4.2	30.0	GoM
10*	M	7	7/23/06	120	1,530	4.4	30.8	GoM
11	F	7	9/13/06	60	1,376	4.6	30.6	GoM
12	M	8	9/13/06	34	751	6.0	29.6	GoM
13	F	8	9/14/06	98	1,072	5.0	29.8	GoM, Str. FL
14	M	7.5	6/07/08	120	1,720	4.4	30.2	East GoM, Str. FL
15	F	7.5	8/29/07	150	1,600	4.4	31.4	Carib., Atl.
16	F	5	8/30/07	90	1,400	4.2	31.0	GoM
17*	M	7.5	8/08/08	97	1,432	4.4	30.2	GoM, Carib.
18*	F	9	8/08/08	3	8	22.0	28.8	Cabo Site
20	F	8	9/08/08	100	1,528	4.4	30.6	GoM, Yuc. Chan.
21	M	7	10/08/08	150	1,560	4.4	31.8	GoM, Str. FL, Carib.
22	F	8	11/08/08	120	1,392	4.4	29.6	GoM
23	F	7.5	7/13/09	180	1,240	4.8	31.0	GoM, Carib.
24	F	7.5–8	7/13/09	109	1,368	4.4	30.6	GoM, Carib., Str. FL
25	M	7	7/16/09	60	1,888	4.2	31.2	GoM
26	F	6.5–7	7/16/09	106	1,008	5.0	30.8	GoM
27	F	7.5	8/28/09	112	1,672	4.4	31.0	GoM
28	F	8	8/29/09	99	1,392	4.4	32.4	GoM, Str. FL
30*	F	8	7/28/10	180	1,408	4.4	31.0	GoM
31	F	7.5	7/28/10	34	80	18.4	30.6	Afuera Site
32	F	7.5–8	7/29/10	35	72	19.8	30.8	Afuera Site
33*	F	8.5	7/29/10	57	116	16.8	30.9	Afuera Site
35	M	8.5	9/23/12	33	928	6.2	30.6	West Carib.
36^F^	M	7.5	5/28/10	90	1,688	4.2	31.6	East GoM, Yuc. Chan.
37^F^	M	6.5–7	6/18/10	56	1,928	4.2	33.0	NE GoM
38^F^	F	7	6/18/10	10	24	21.8	32.8	North of FL Site

* Tag recovered

^F^ Tagged off west Florida coast

^†^ GoM = Gulf of Mexico; Carib. = Caribbean Sea; Str. FL = Straits of Florida; Yuc. Chan. = Yucatan Channel

### Whole Archive Depth-Temperature Profiles

**Fig 3:**
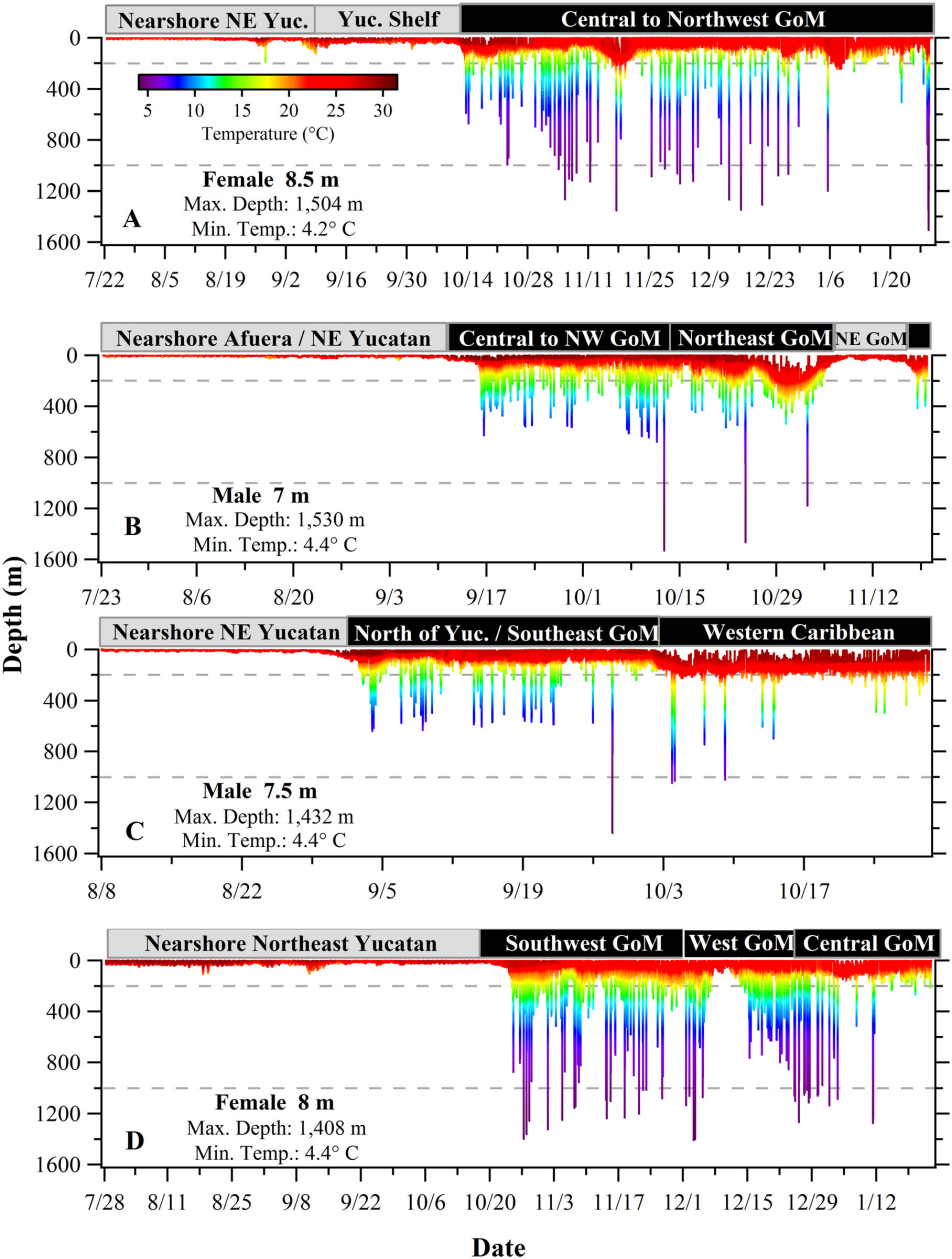
Depth-temperature profiles from recovered PSATs deployed on whale sharks (*Rhincodon typus*) off the Yucatan Peninsula, Mexico. (A) The 190-day track of Shark 9. (B) The120-day track of Shark 10. (C) The 82-day track of Shark 17. (D) The 180-day track of Shark 30. Based on their most probable tracks, the approximate geographic area is indicated within the grey (continental shelf) and black (offshore) boxes above the profiles. Broken lines delineate the epipelagic, mesopelagic, and bathypelagic zones.

The complete depth-temperature profiles from the four longest high-resolution data sets (Sharks 9, 10, 17 and 30; ranging 82–190 days) showed a distinct post-tagging inshore phase with the sharks remaining off the northeast Yucatan Peninsula for a period ranging 3–12 weeks. This was followed in Sept-Oct by an offshore phase away from the tagging area, characterized by regular descents into deeper water and variable patterns of habitat utilization depending on the geographic area and/or the available vertical habitat (Fig 3). Although most vertical movements were confined to the epipelagic zone (0–200 m), when in oceanic waters, tagged whale sharks of both sexes exhibited a pattern of frequent but relatively short duration dives into the mesopelagic zone (200–1,000 m) and occasional dives extending briefly into the bathypelagic zone (>1,000 m) (Fig 3). Although all four sharks spent time at the surface or near-surface, Sharks 10 and 17 (Fig 3B and 3C) occasionally remained well below the surface for extended periods of time. Most notably, Shark 10 stayed below 50 m depth for 75 continuous hours from mid-day October 30 through late afternoon November 2, 2006. We did not find any conspicuous weather phenomena (in the form of named storms) in the GoM and Caribbean Sea that might explain the apparent avoidance of the surface during these periods (source:http://www.wunderground.com/hurricane/hurrarchive.asp).

### Time-at-Depth and Temperature

**Fig 4:**
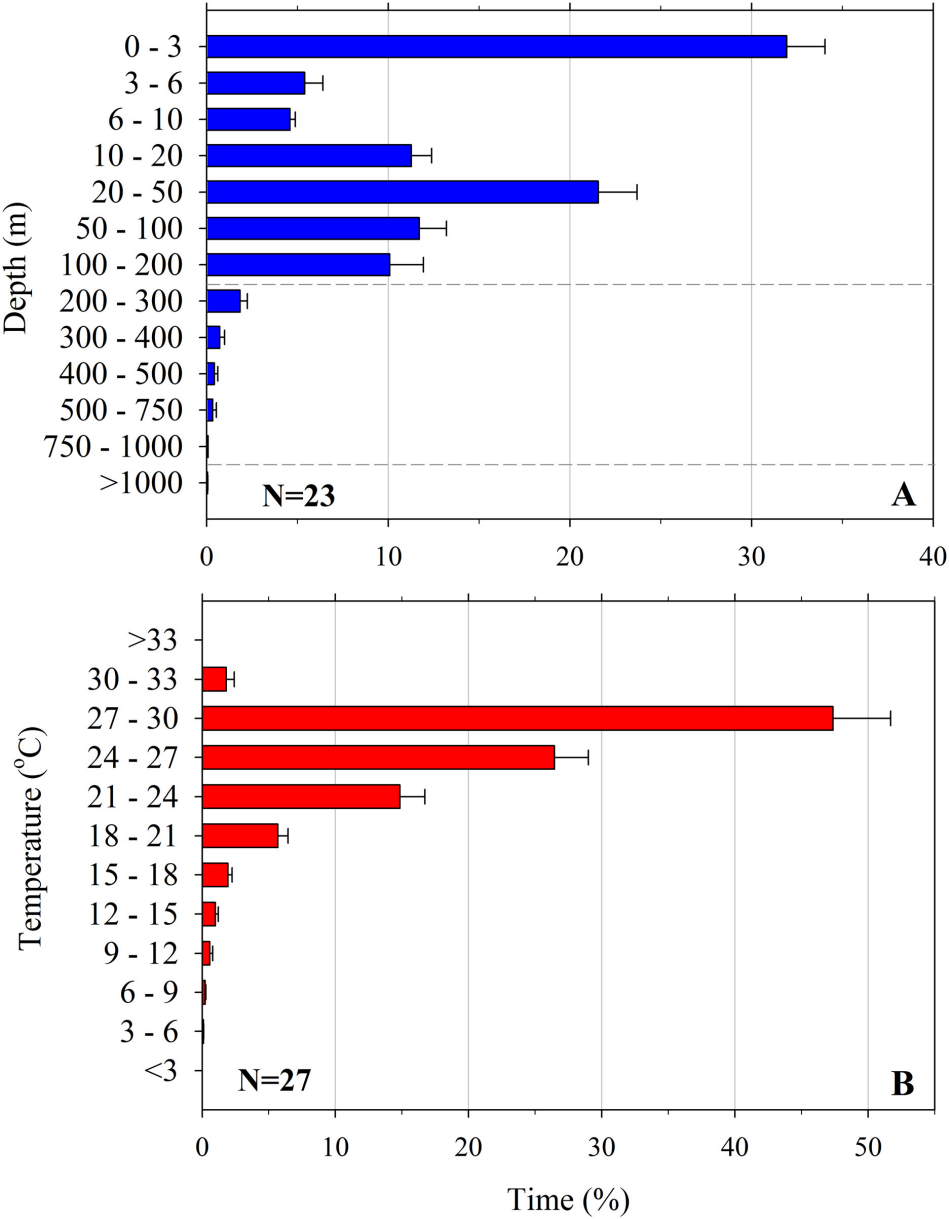
Composite histograms for satellite-tagged whale sharks (*Rhincodon typus*) off the northeast Yucatan Peninsula and west Florida. (A) Time-at-depth. (B) Time-at-temperature. Values are mean ±SE. Broken lines in A delineate the epipelagic, mesopelagic, and bathypelagic zones.

Tagged sharks spent 31.9% of their time in the 0–3 m depth range and 21.6% of time in the 20–50 m range. A total of 96.5% of time was spent in the epipelagic zone, 3.40% in the mesopelagic zone, and 0.06% in bathypelagic depths (Fig 4A). We found no time-at-depth differences between the sexes (K-S test p = 0.898) or when comparing large (>7.5 m TL) to small (≤7.5 m TL) sharks (K-S test p = 0.898). Pooled time-at-temperature data (N = 27) indicated the sharks spent the largest proportion of their time (47.4%) in water ranging 27–30°C and spent only 0.9% of their time in water ≤12°C (Fig 4B). On average, the time spent in waters of various temperatures did not differ significantly by sex (K-S test p = 0.997) or by size (K-S test p = 0.833).

### Day vs Night Depth and Temperature

**Fig 5:**
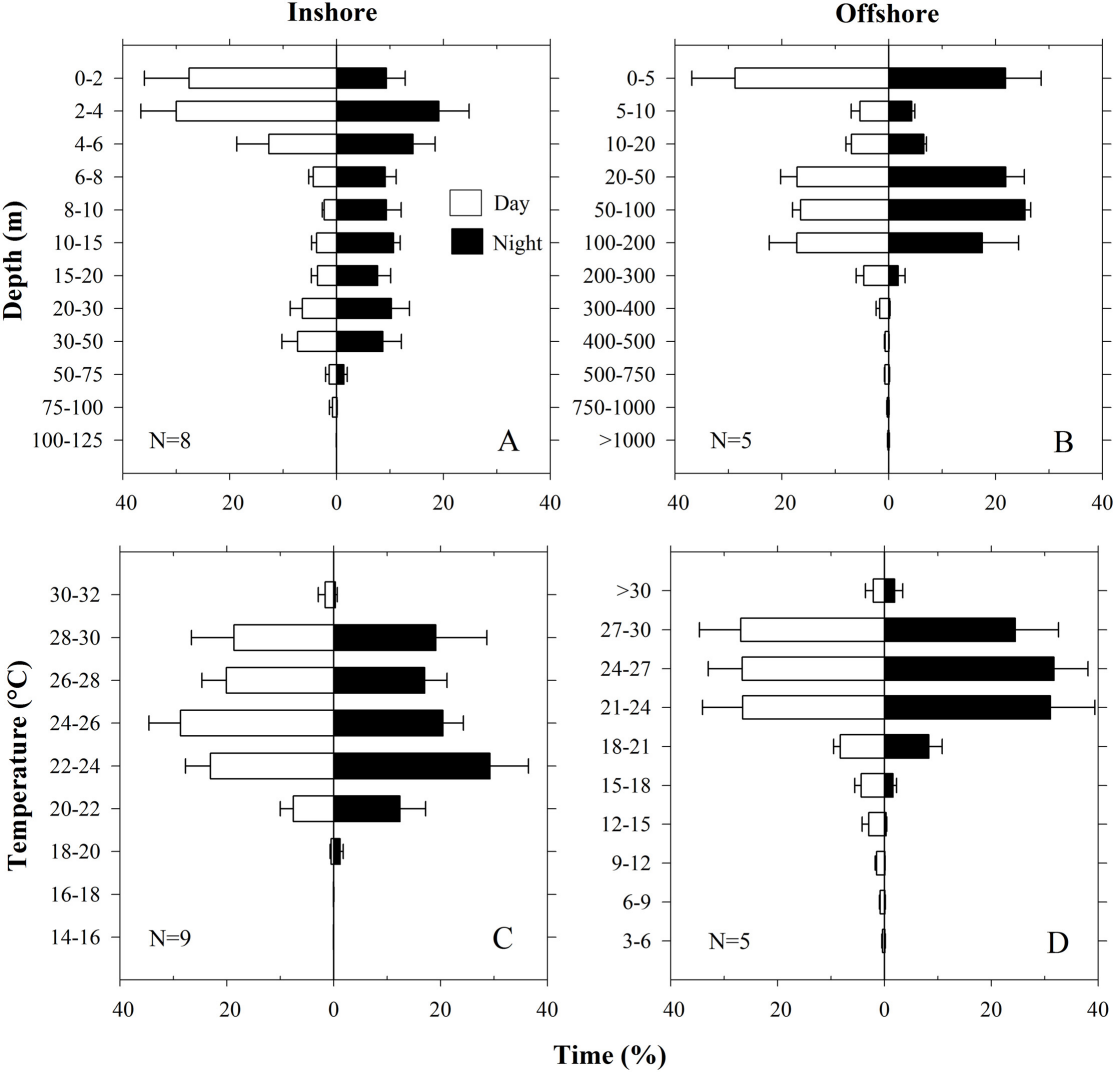
Composite histograms from high resolution data of recovered tags comparing day vs night depth and temperature during inshore and offshore phases. (A) Time-at-depth inshore. (B) Time-at-depth offshore. (C) Time-at-temperature inshore. (D) Time-at-temperature offshore. Error bars represent SEM.

Data from recovered PSATs enabled us to precisely distinguish day from night using recorded light levels and in doing so identify diel patterns of vertical movement. Based on the MPTs and depth profiles, we further separated the inshore and offshore phases of the tracks to investigate patterns of habitat utilization (see Fig 3 for approximate inshore/offshore delineation). For the inshore phase, the mean daytime depths for nearly all individuals were significantly shallower than nighttime depths (Table 2; t-test p = 0.0000). The only exception was Shark 4 which showed no significant difference (p = 0.1155); however, its inshore phase was less than 1 day long. An inshore time-at-depth composite analysis (N = 8) failed to detect a significant difference but further demonstrated a skewed depth distribution with 57.6% of daytime hours spent in surface waters (0–4 m) compared to 28.5% of night hours in this depth range (K-S test P = 0.099; Fig 5A). For the offshore phase, a reversal of this diel pattern was noted with mean daytime depths for individuals significantly deeper than the nighttime depths (Table 2; t-test p = 0.0000). Although the offshore time-at-depth composite analysis (N = 5) showed no significant difference between day and night (K-S test P = 0.536; Fig 5B), it did reveal the deepest diving activity occurred predominantly during daylight hours with 8.1% of time spent at ≥200 m during the day compared to 2.3% at night (Fig 5B). This diel pattern was similarly reflected in day vs night time-at-temperature profiles. During the inshore phase the sharks spent 31.1% of their time during the day in temperatures <24°C compared to 43.0% during the night at these cooler temperatures (Fig 5C). When offshore, sharks spent 5.4% of time during the day in water <15°C compared to 0.7% spent during the night at these colder temperatures (Fig 5D). However, these day vs night distributions were not found to be significantly different for either the inshore (K-S test P = 1.000) or offshore phases (K-S test P = 0.787).

**Table 2:** Statistical results of day vs night comparison of whale shark mean depths from recovered PSAT tags. The inshore and offshore phases of their tracks were analyzed separately.

INSHORE					OFFSHORE			
Shark No.	Days of Data	Day Depth (m) Mean±SE	Night Depth (m) Mean±SE	P value	Days of Data	Day Depth (m) Mean±SE	Night Depth (m) Mean±SE	P value
4	1	1.9±0.14	2.4±0.16	0.1155	30	60.4±0.58	36.8±0.28	0.0000
6	2	2.3±0.04	5.4±0.10	0.0000	0	-	-	-
7	18	3.1±0.01	6.0±0.02	0.0000	0	-	-	-
9	83	12.9±0.04	16.6±0.04	0.0000	107	44.1±0.22	41.6±0.15	0.0000
10	52	3.0±0.01	5.6±0.02	0.0000	68	74.0±0.35	67.2±0.21	0.0000
17	24	6.7±0.01	8.2±0.01	0.0000	58	95.2±0.10	89.5±0.06	0.0000
18	3	4.3±0.01	8.8±0.03	0.0000	0	-	-	-
30	88	9.9±0.01	16.1±0.01	0.0000	92	58.9±0.10	43.9±0.06	0.0000
33	57	15.4±0.02	20.7±0.02	0.0000	0	-	-	-

### Dynamic Patterns of Movement and Diel Vertical Migration

**Fig 6:**
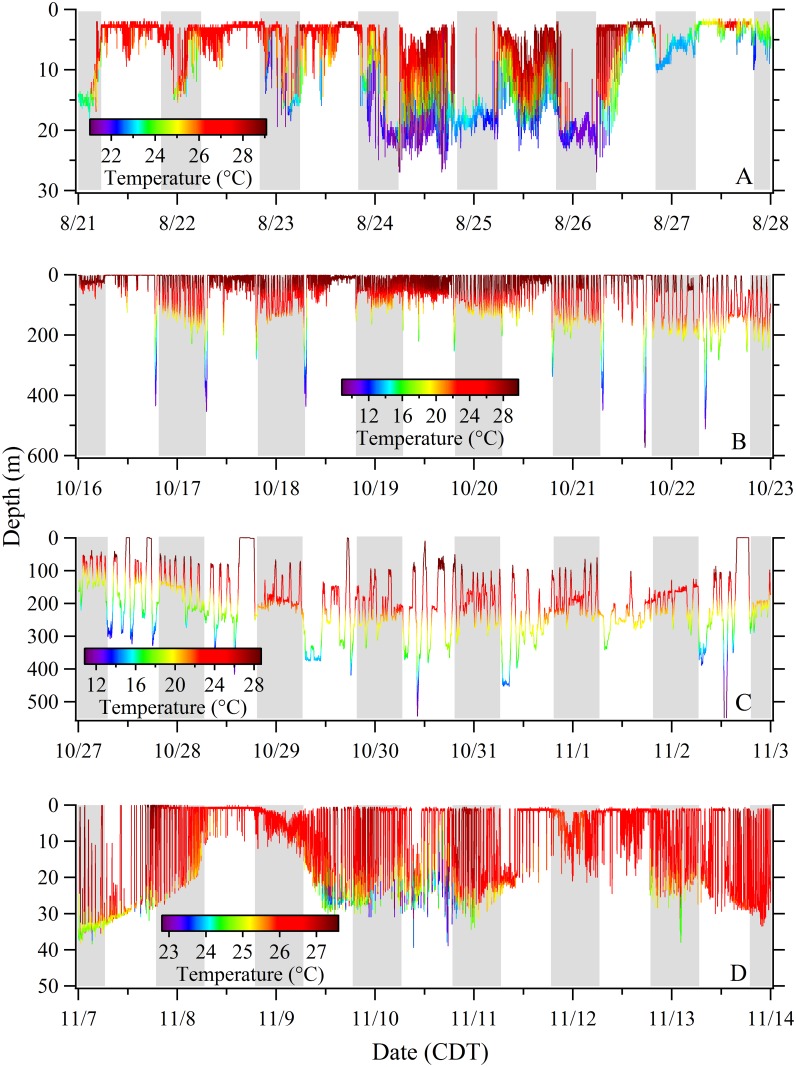
Dynamic patterns of vertical movement during four week-long periods of the track of Shark 10. Variable patterns can be observed including: (A) reverse diel vertical migration; (B) crepuscular deep dives; (C) extended periods of low frequency oscillations at depth; and (D) near surface high frequency vertical oscillations. Vertical grey bars indicate nighttime. The recovered PSAT provided measurements at 30 second intervals.

**Fig 7:**
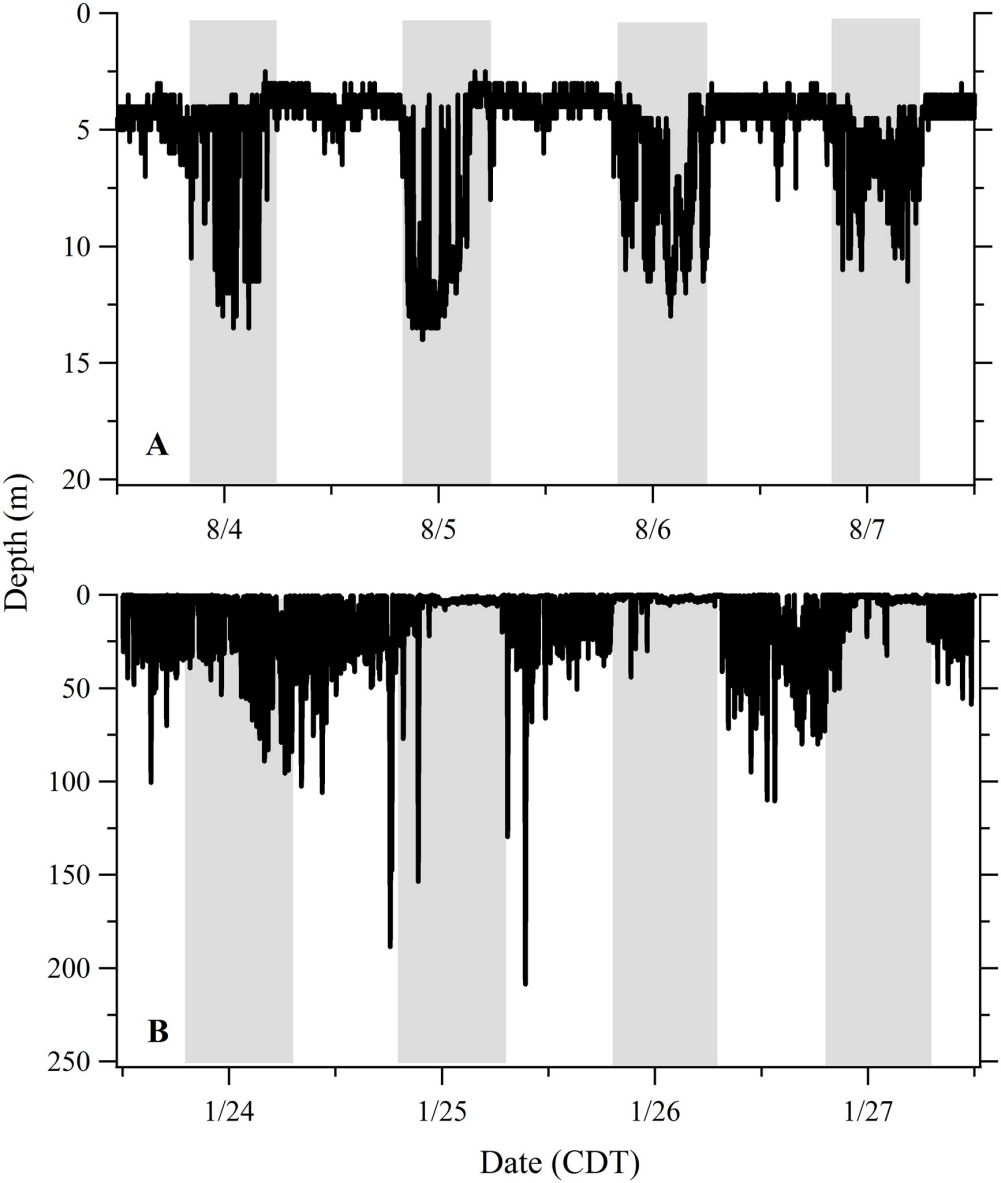
Changes in the diel vertical migration (DVM) pattern for Shark 9. (A) Typical reverse DVM demonstrated inshore near the northeast Yucatan Peninsula tagging site. (B) Contrasting pattern of protracted surface time at night observed offshore in the central Gulf of Mexico, north of the Yucatan Peninsula.

**Fig 8:**
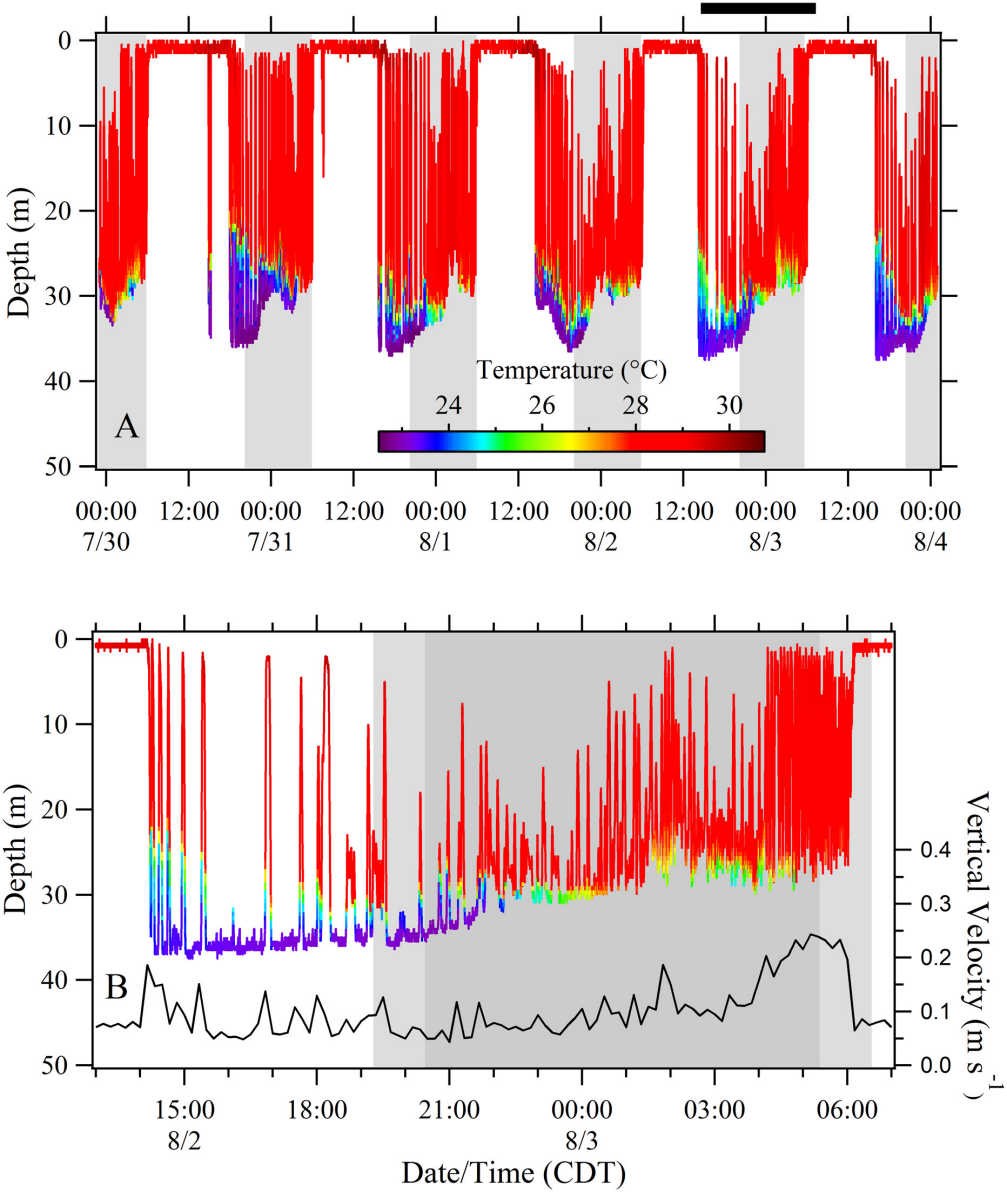
Depth-temperature profiles for Shark 33 tagged in the Afuera aggregation. (A) Five-day period post-tagging demonstrating a pattern of daytime surface swimming beginning near sunrise followed by regular vertical movements beginning in the mid afternoon. (B) An 18-hour period demonstrating dynamic vertical oscillations beginning in the afternoon and ending near sunrise (period corresponds to the black bar in A). High frequency bounce dives were observed during the 2-hr period prior to the resumption of surface swimming (04:00–06:00). The absolute vertical velocity increased to approximately 0.25 m s^-1^ during this period. The dark grey shading represents nighttime while the lighter gray represents twilight.

Dive profiles from recovered PSATs provided a high resolution picture of dive behavior that was useful in identifying dynamic patterns in vertical habitat utilization. To illustrate this variability, we plotted four week-long dive profiles from the 120-day track of Shark 10 (Fig 6) during which time the shark moved from the inshore area of the northwest Yucatan Peninsula, through the central GoM, then remained in the shelf edge waters of the northern GoM (Figs 1A and 3B). Highly variable patterns of vertical movement can be observed including examples of reverse diel vertical migration (DVM), crepuscular deep dives, extended periods of low frequency oscillations at depth, and higher frequency near-surface oscillations, among others (Fig 6). Although reverse DVM was commonly observed, we noted contrasting periods where the sharks largely remained in surface waters during the night and undertook regular vertical oscillations during the day (Fig 7). Sharks feeding in the summertime Afuera aggregation typically demonstrated a pattern of swimming at or near the surface during the day and then abruptly changing to a sub-surface pattern of vertical movements beginning in the mid-afternoon (reverse DVM; Fig 8A). This surface swimming pattern was most commonly initiated at sunrise although we noted exceptions where surface feeding commenced in the middle of the night. During this particular 5-day example (Fig 8A), the vertical movements initiated in the early afternoon extended to depths below the thermocline (~30 m) but then transitioned into a pattern where the dives stopped above the thermocline by the middle of the night. The surface feeding pattern resumed again the next day near dawn and continued for period of 8 to 11 hours. During the first few hours sub-surface, the sharks largely remained in colder water (~23°C) but also performed sporadic forays toward the warmer surface waters (~28°C; Fig 8B). The forays typically became more frequent later in the night and then rapid vertical oscillations were commonly observed during the 1–2 hour morning period immediately prior to twilight and the resumption of surface swimming (Fig 8B). In this example, the period of pre-sunrise oscillations (04:10–06:00) had an overall increase in absolute vertical velocity ($\bar{x}$ = 0.22 m s^-1^) and included bounce dives with mean ascents (0.31 m s^-1^) significantly faster than descents (0.22 m s^-1^; t-test p = 0.000). Comparable dives prior to this period (00:00–04:00) were more variable with mean ascent rates (0.19 m s^-1^) not significantly different from descents (0.18 m s^-1^; t-test p = 0.439). For Shark 33, this distinct reverse DVM pattern continued until late August when it gradually transitioned into a pattern of nearly continuous vertical oscillations that formed V-shaped profiles spanning both day and night periods (S1 Fig).

### Fast Fourier Transforms

**Fig 9:**
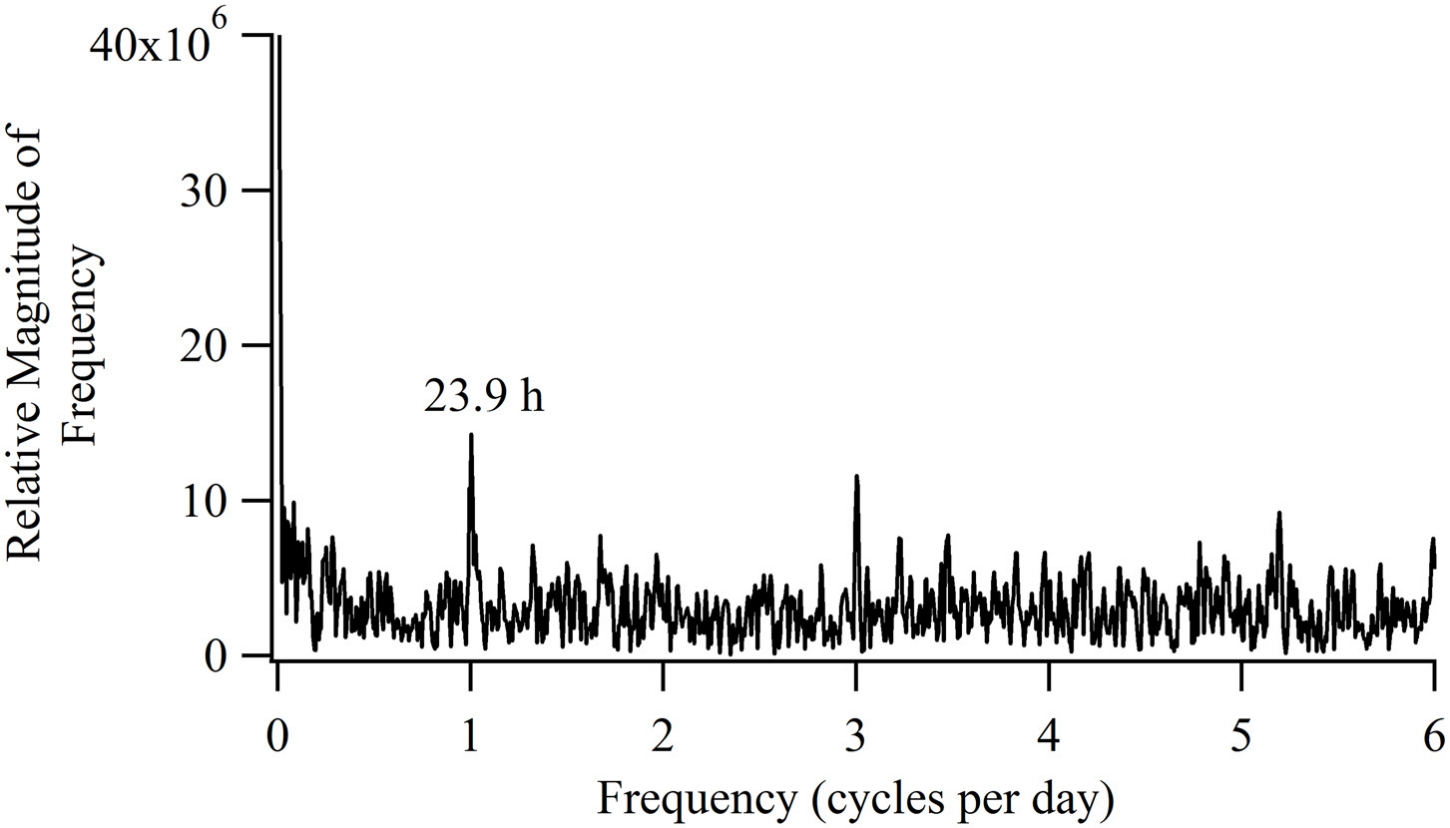
Fast Fourier transform-generated periodogram for depth data of Sharks 30. The high amplitude peak at 1 cycle day^-1^ is indicative of a diel rhythm in the vertical movements of the shark.

We generated FFT periodograms from the high resolution data sets to detect periodic signals in the depth records. For all the sharks, the dominant periodic component in the vertical movements was 1 cycle day^-1^ (a diel rhythm) as exemplified by Shark 30 (Fig 9). Smaller spectral peaks at multiples of 1 cycle day^-1^ were also observed but most likely represented harmonics of the true diel component [27]. In all cases, the main spectral peaks were identified with and without the ‘hamming’ window function, indicating these periodicities were not artifactual [28]. Discrete sub-sections of the different data sets were analyzed with the FFT separately (e.g. inshore separate from offshore) but no other prominent peaks were detected. The FFT analyses of depth did not detect evidence of a correlation with lunar cycle.

### Crepuscular Pattern of Deep Dives

**Fig 10:**
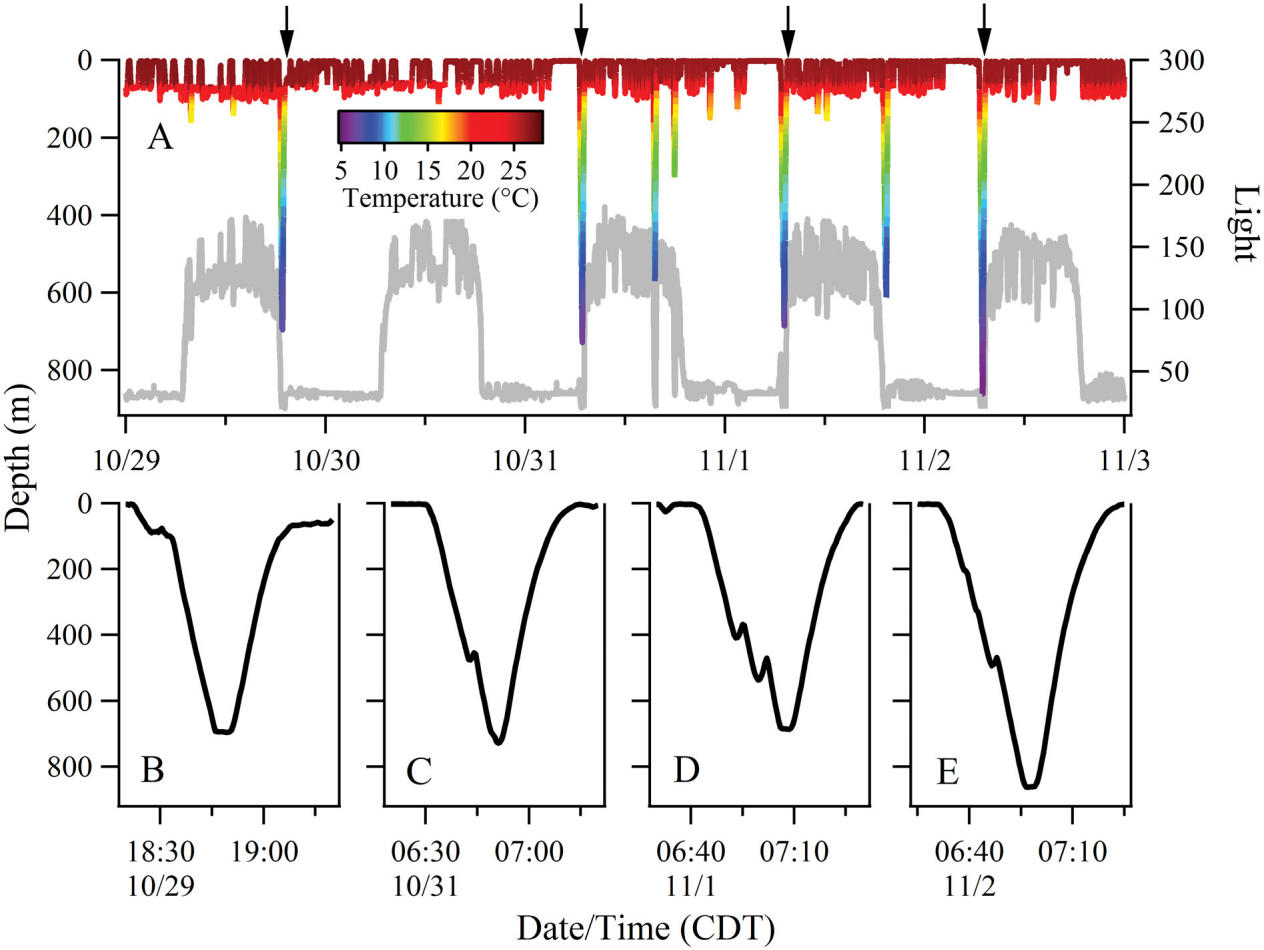
Crepuscular pattern of extreme dives observed in Shark 9 while in the central Gulf of Mexico. (A) Depth-temperature profiles during the period of October 29 through November 2. Tag measured light levels are shown as gray trace. (B-E) Close-up depth profiles of extreme dives coinciding with sunrise or sunset (marked with arrows in A). Note the stutter steps during descent at depths of 400–500 m.

Data from recovered PSATs revealed that 32% of the whale shark’s isolated deep dives (>200 m) were occurring around the times of dusk and dawn. In our example of Shark 9, the crepuscular dives were to depths as much as five times the depth of the dive activity occurring before or after dawn and dusk (Fig 10). These dives had V-shaped depth profiles with descents that often had brief changes in vertical direction (or stutter steps; Fig 10B and 10E).

### Extreme Dives

**Fig 11:**
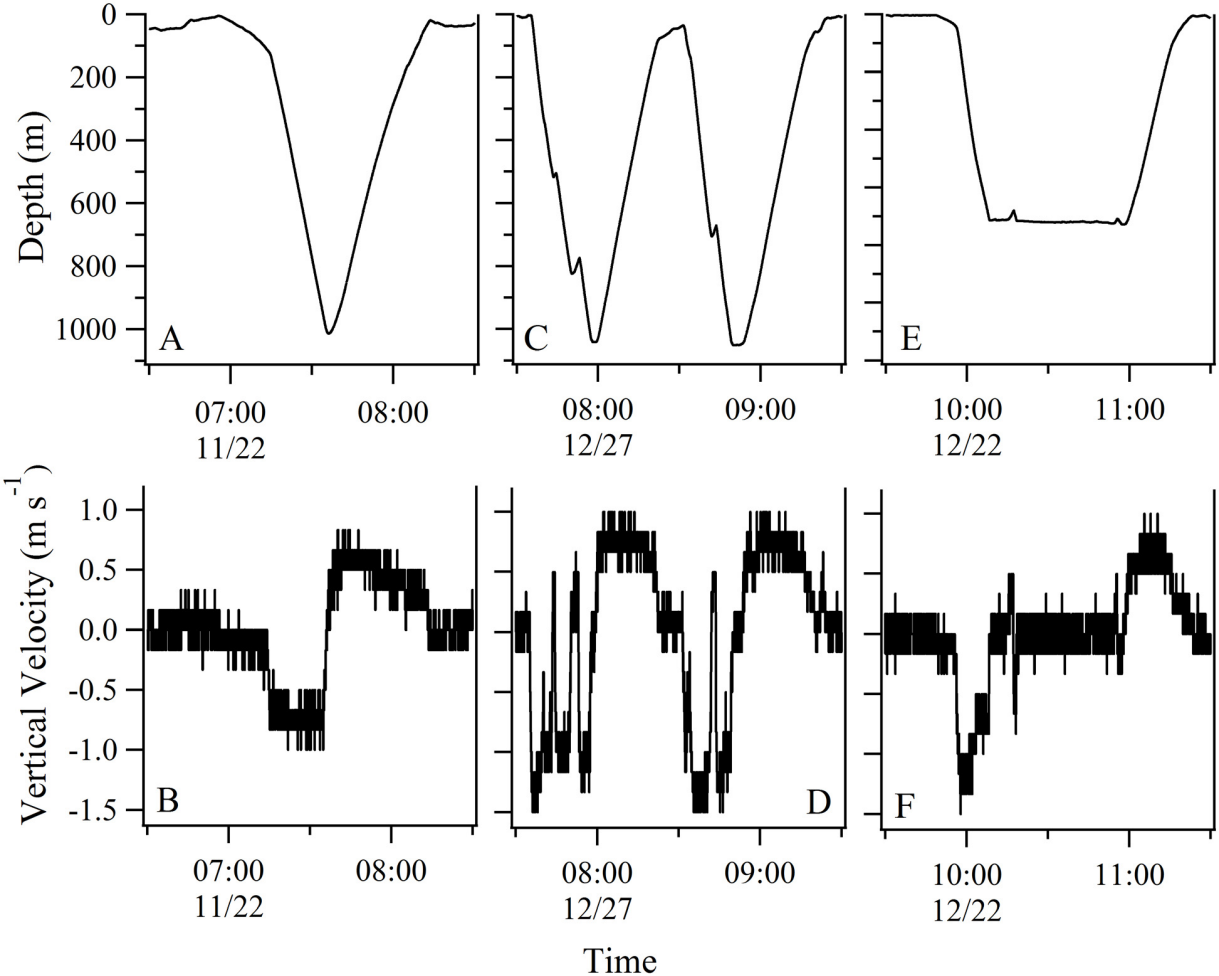
Profiles of extreme dives (>500 m) and vertical dive velocities from Shark 30. (A) Time-depth profile for a V-dive. (B) Consecutive V-dives with stutter steps on descent. (C) U-dive. (D-F) Corresponding rates of vertical movement (m s^-1^) during these same dives. Data were recorded by the tag at 3 second intervals.

Deep dives (>500 m) most commonly had V-shaped time-depth profiles (Fig 11A). To better characterize these extreme dives, we examined the rates of vertical velocity during individual dives (Fig 11B). For this comparison we selected dives with descents initiated at or near the surface, maximum depths of at least 500 m, and ascents that were initiated almost immediately after reaching the dive’s maximum depth. We included 57 dives that fit this profile from five sharks with recovered tags (Sharks 4, 9, 10, 17, and 30). The mean absolute rate of descent (0.68 m sec^-1^) was significantly faster than the mean ascent rate (0.50 m sec^-1^) (paired t-test; p < 0.0005). The highest instantaneous rates of vertical velocity measured during any of the extreme dives were -1.83 and 1.50 m sec^-1^ for descent and ascent, respectively (Shark 17). Extreme dives that did not fit this V-profile included those with stutter steps on the descent (Fig 11C and 11D). We found that 46.2% of the extreme dives from these five sharks had some form of distinct stutter step in the descent whereas only 1.2% showed a comparable stutter during ascent. The stutter steps occurred at depths of 107–1,041 m and mean depth of 475 m. Less commonly observed were U-dives where the shark descended to extreme depth but remained at depth for an extended period of time before ascending to the near surface (Fig 11E and 11F).

## Discussion

In this research we characterize the vertical habitat of whale sharks in the GoM using one of the largest satellite tagging studies of this species. Our results underscore the whale sharks’ close association with near-surface water and reveal details of their habitat use when in deeper water off the continental shelf. Brief forays into the bathypelagic zone are not uncommon and *R*. *typus* is capable of descents to at least 1,928 m, the deepest recorded dive we are aware of for any whale shark. We show that *R*. *typus* can remain continuously at depth (>50 m) for more than three days, suggesting they are not obligate surface feeders (Figs 3B and 6C). Through the physical recovery and direct download of nine PSAT tags, our study provides detailed, long-term depth profiles revealing highly complex and variable vertical movement patterns of whale sharks.

### Reverse Diel Vertical Migration

When in coastal, relatively shallow water, whale sharks in our study showed a reverse DVM pattern, with greater time near the surface during the day and more time at depth during the night (Figs 5A, 7A and 8A), a pattern seen in other studies of *R*. *typus* in the Indian Ocean [13,15]. When offshore, individual sharks in our study deviated from this pattern and demonstrated greater plasticity in vertical habitat selection. The deepest offshore dives occurred primarily during the day or twilight hours (Figs 5B and 10A), a pattern documented in several other *R*. *typus* studies [13,15,16,17]. Diel rhythms in vertical activity have been reported in a number of shark species but more commonly follow a deeper-by-day and shallower-by-night pattern (i.e. normal DVM) [24,31–34], often a characteristic of pelagic visual predators foraging on prey within the deep scattering layer (DSL) [35]. However, normal DVM also has been observed in the filter-feeding megamouth *Megachasma pelagios* [36] and basking sharks *Cetorhinus maximus* [37] in response to the circadian rhythms of their zooplankton prey. In our study, exceptions to the typical reverse DVM pattern were common. For example, we noted that individual whale sharks can remain well below the surface (>50 m) continuously for several days and can abruptly change their use of vertical habitat (Fig 6) or gradually transition from one pattern to the next (S1 Fig). Depending on habitat type and zooplankton behavior, *C*. *maximus* can similarly shift from normal DVM to reverse DVM, likely in response to the reverse DVM of its prey (*Calanus* spp.) [37]. Although none of the sharks in the present study demonstrated what can be called normal DVM, we did note contrasting periods where sharks remained almost continuously near the surface at night while in offshore waters (Fig 7). Since whale sharks, like basking sharks, are adept at finding dense patches of prey [38–40], these pattern shifts are most likely related to maximizing foraging efficiency when encountering changes in environmental, bathymetric, and/or food availability conditions.

### Inshore Patterns of Vertical Movement

When inhabiting the inshore Afuera aggregation area off the Yucatan Peninsula, whale sharks shifted at dawn from a pattern of sub-surface vertical movements to a distinct surface swimming behavior, to initiate feeding (Fig 8A). At Ningaloo Reef, Western Australia, data loggers revealed that ram surface feeding activity of whale sharks peaked at sunset and that this change in behavior was in anticipation of dense patches of tropical krill (*Pseudeuphausia latifrons*) that form at that time [39]. In our study, the whale sharks in the Afuera aggregation do not surface feed on vertically mobile prey but rather on dense homogenous patches of buoyant fish eggs from the little tunny [20]. A single female *E*. *alletteratus* can release as many as 1.75 million eggs in several batches during a spawning season that peaks during the warmest months [41], the same time whale shark abundance peaks in the Afuera [20]. Nothing is known of the courtship and spawning behavior of *E*. *alletteratus* but spawning reportedly occurs between midnight and dawn in the Atlantic Bluefin tuna (*Thunnus thynnus*) [42]. We hypothesize the little tunny spawning activity in this area is occurring in the middle of the night and/or the morning hours and the whale sharks’ shift to surface waters is linked to the timing of these spawning events. In the northern GoM, whale sharks were similarly observed surface feeding during morning hours on recently spawned (mid-embryonic stage) little tunny eggs [43]. These authors also noted adult *E*. *alletteratus* in spawning condition and suggested that the spawning activity was occurring at the whale shark aggregation site. Our observation that surface feeding occasionally resumed in the middle of the night suggests variability in the timing of tunny spawning events. The period of high frequency oscillations (or bounce dives) just prior to sunrise showed an increase in absolute vertical velocity ($\bar{x}$ = 0.22 m s^-1^; Fig 8B). This is very similar to the peak in whale shark vertical movement (~0.25 m s^-1^) observed prior to sunset at Ningaloo Reef [39]. These authors attributed this peak in vertical activity, along with increased ascent pitch angles, to a vertical search of the water column in anticipation of the formation of dense swarms of krill. The whale sharks in the GoM and northwestern Caribbean Sea may similarly be attempting to relocate the densest prey patch, perhaps by locating the spawning fish themselves as we have similarly observed little tunny in the vicinity below the surface-feeding whale sharks in the Afuera aggregation. Other late afternoon/nighttime forays toward the surface (Fig 8B) may represent a vertical search pattern for sub-surface planktonic prey, although we have no data from sub-surface plankton tows to substantiate this.

After this protracted period of daytime surface swimming, an abrupt change occurs whereby the sharks initiate a sub-surface swimming pattern extending below the thermocline with occasional upward forays toward the surface during the first few hours after initiation (Fig 8A and 8B). Later into the night, the vertical oscillations became more frequent but extended to a point just above the thermocline. It is possible this pattern represents a form of behavioral thermoregulation whereby the sharks are dissipating heat they have acquired during the protracted time in warm surface waters. Off Western Australia, Thums and her colleagues (2013) [44] demonstrated evidence of behavioral thermoregulation in *R*. *typus* but for the purpose of warming rather than cooling. These authors showed that post-dive surface duration was negatively correlated with the minimum temperature of dives and hypothesized that thermoregulation was a major factor driving a protracted surface interval after spending time in cool, deep water. In our Afuera tagging area, a surface ram filter-feeding whale shark could be particularly prone to overheating given the massive volumes of warm water (~29°C in the Fig 8A example) passing across its gills. During this type of surface swimming, the dorsal surface of the sharks’ head and part or all of the dorsum between the rostrum and the first dorsal fin are exposed [21], potentially subjecting the brain to damaging high temperatures. Given the whale sharks’ dark color and intensity of the summer sun in the Mexican tropics, solar heating may also contribute to the need for thermoregulation after extended periods at the surface. However we cannot discount alternative or perhaps additive explanations for these observed behaviors. Surface plankton tows in the Afuera site have estimated a fish egg biomass of 21.1 g m^-3^ [20]. At this concentration and a filtering rate of 614 m^3^h^-1^ [21], a 6.2 m TL whale shark surface feeding for 11 hours would ingest 142.5 kg of fish eggs equating to about 43,000 Kcal, an energy content 6 to 10 times greater than the estimated daily ration for comparably sized wild and captive whale sharks [20,21]. Given this abundance of fish eggs, the sharks may be abruptly stopping their surface feeding in the afternoon due to satiation. The subsequent sub-surface patterns of movement to colder water could function to decrease metabolism during a non-feeding period. Shifting to cooler water after feeding could be advantageous by maximizing energy uptake. Studies with the Atlantic stingray (*Dasyatis sabina*) have indicated that shifting into lower temperature water decreased evacuation rates which resulted in a significant increase in total absorption [45]. In the adult male dogfish (*Scyliorhinus canicula*), the “hunt warm, rest cool” strategy was estimated to lower daily energetic costs by just over 4% [46]. It is possible the whale sharks in this type of tropical habitat benefit by using a form of post-feeding thermotaxis to improve digestive uptake.

### Extreme Diving (>500 m)

When in bathymetrically non-constraining habitat, whale sharks undertook regular descents into mesopelagic and bathypelagic depths usually followed by nearly immediate ascents (Fig 3). Although *R*. *typus* has been shown to dive to depths in excess of 1,000 m [10,13], time-depth profiles of these extreme dives have seldom been examined in detail and the function of these dives remains unclear. Brunnschweiler and his colleagues (2009)[13] suggested the most likely explanation was a search behavior for feeding opportunities but also stated that the acquisition of navigational cues (e.g. magnetic gradients) could explain the deep dives by *R*. *typus*. In our study, nearly one third of the observed extreme dives closely coincided with dawn or dusk (Fig 10). Off Western Australia, Wilson et al. (2006)[15] similarly reported that deep dives by whale sharks had a crepuscular pattern and suggested that deep bounce dives at times of light transition could serve to locate vertically migrating prey during a brief window of vulnerability. Fatty acid profiles from *R*. *typus* stomach contents also suggest these animals forage in meso- and bathypelagic zones in the Indian Ocean [47]. The fact that most of the deep diving activity in our study occurred during the day supports the feeding hypothesis, since the DSLs are typically more pronounced during the day when many vertically migrating species are concentrated at depth [48]. If searching for food was the primary function of deep dives, it is reasonable to assume the sharks would occasionally level off and take advantage of a layer of prey for some period of time. Devil rays (*Mobula tarapacana*) off the north central Atlantic that dive to nearly 2,000 m exhibited step-wise ascents that were consistent with the hypothesis that the rays were feeding on prey in high-density layers at depth [49]. Although we did not observe any distinct stepwise vertical movements at depth, we did note brief changes in vertical direction or stutter steps in many of the descents during both crepuscular (Fig 10) and non-crepuscular dives (Fig 11). Overall, nearly half of the extreme dive descents had stutter steps and they occurred at a mean depth of 475 m. In a study from the northern GoM, the DSLs were located at variable depths in this region but the most prominent layers were consistently found at daytime depths of 450–550 m from the surface [50]. We hypothesize that the stutter steps observed on extreme descents represent brief but intense foraging events on concentrated mesopelagic zooplankton and micronekton. Similarly, the protracted bottom time during deep U-dives (Fig 11E) could represent a foraging period on demersal macrozooplankton.

Crepuscular deep dives could also function as an aid to navigation. In a study tagging southern bluefin tuna (*Thunnus maccoyii*) with archival tags, a comparable pattern of crepuscular deep dives was revealed where these fish made “spike dives” at sunrise and sunset [51]. These authors found the dives were precisely timed and occurred at an almost identical point in the sun’s elevation. This point coincides with when the earth’s magnetic field intensity reaches its maximum. They proposed that the tuna, through the use of their pineal apparatus, were diving down at that time to get a better read of their “magnetic map.” In our example of deep crepuscular dives from Shark 9 (Fig 10), the MPT indicates the shark was in the central GoM in about 2,000–3,000 m water at that time (Fig 1A). Using their electrosensory system, sharks have the ability to detect magnetic fields [52] and it is widely accepted that they use the earth’s geomagnetic field for orientation and navigation [53]. Because the geomagnetic intensity gradient increases with depth [54], observations of tracked sharks moving periodically to significant depths has been theorized as a behavioral means of improving their magnetic read [55,56]. Taken together, it is plausible the whale sharks’ crepuscular deep dives or extreme dives in general may play a navigational role for these ocean travelers.

The glide hypothesis for whale sharks, supported via accelerometry data from relatively shallow dives at Ningaloo Reef (<100 m) [18], may also help explain the function of these extreme dives. Based on time-depth profiles, the deep dives of the reef manta ray were similarly attributed to powerless glides as an energetically efficient means of horizontal travel [57]. However, in both the aforementioned studies, the descents were slower than the ascents. The V-profile extreme dives in our study (Fig 11) were characterized by faster descents (0.68 m sec^-1^) than ascents (0.50 m sec^-1^), a pattern associated with prey-searching behavior in predatory sharks [58]. These rates of movement are considerably faster than the mean vertical velocities reported for isolated V-dives at Ningaloo (0.16 m sec^-1^ and 0.23 m sec^-1^ for descent and ascent, respectively) [18]. Whether these differences have a bearing on the function or not must await future studies using tags that can quantify three-dimensional movement during deep dives.

### Conclusions and Significance

Sustainable conservation and management strategies for shark populations necessitates detailed knowledge of their spatial ecology [59]. In this study we examined the vertical movement data from 31 whale sharks tagged off the Yucatan Peninsula and west Florida with PSAT tags to better understand their sub-surface behaviors in space and time. Our results demonstrate dynamic patterns of habitat utilization likely in response to changing biotic and abiotic conditions that influence the distribution and abundance of the sharks’ prey. Recent studies indicate that whale shark foraging is not species-selective but instead targets high biomass prey patches of various forms [39,40]. The precise cues the sharks use to detect the densest prey patches requires further investigation, but captive studies provide evidence that the olfactory sense of *R*. *typus* may be involved in detecting prey-associated chemicals [60].

When in oceanic waters, whale sharks appear to utilize mesopelagic and bathypelagic habitats for foraging but the possibility that deep diving behavior also aids in navigation and energy-efficient horizontal transport is worthy of further study. Tagging with additional sensors, such as accelerometers, can provide data to better interpret the function of vertical movement patterns of free-swimming whale sharks [39]. However, assessing oceanic behaviors still presents challenges as accelerometers at present are used for short deployments where tags can be readily recovered due to memory constraints and/or bandwidth limits of Argos service [61]. For scenarios where the recovery of tag packages is realistic, the synergistic approach of concurrently deploying an accelerometer, depth sensor, and camera with a light source has proven to be a viable means of confirming the function of behaviors of large, difficult to observe species [62].

The northeast Yucatan Peninsula is the site of one of the largest and most important feeding aggregations of whale sharks known to science [8] but is at risk from negative impacts from human activities [20]. Expansion of the protected areas for whale sharks, associated species, and their habitats in this region is recommended. The daytime surface feeding habits of sharks in the Afuera aggregation, which overlaps with ecotourism activities [20], highlights the need for policy makers to limit the industry to avoid significant disruption of the sharks’ natural behaviors. Protracted surface feeding periods off the northeast Yucatan, an area with significant commercial shipping activity [63], makes these sharks vulnerable to vessel strikes and underscores the need for enhanced shipping regulations in this hotspot of marine biodiversity [64]. On a broader scale, relatively rare plankton specialists like the whale shark are potentially more vulnerable to the effects of climate change than other pelagic species [65]. Furthermore, assessing the threats to this species from marine pollution, including deep water petroleum leaks and spills, and from fishing gear set at varying depths in the water column, requires us to know not only the horizontal movements of whale sharks but their vertical movements as well [64]. Advancing our understanding of the drivers of movement and habitat selection in the whale shark, therefore, is essential for developing effective conservation measures and predicting the impacts of environmental change on this species.

## Supporting Information

S1 Fig Depth-temperature profile for Shark 33 demonstrating a shift in vertical movement pattern. The reverse diel vertical migration transitions with a progressive decrease in daytime surface time and increase in vertical oscillations forming V-shaped profiles that span both day and night.
(TIF)
