# Supplementary figures and images for: Vertical Movements and Patterns in Diving Behavior of Whale Sharks as Revealed by Pop-Up Satellite Tags in the Eastern Gulf of Mexico
Source: PLoS One. 2015 Nov 18;10(11):e0142156. doi: 10.1371/journal.pone.0142156 (PMC4651344; doi:10.1371/journal.pone.0142156)

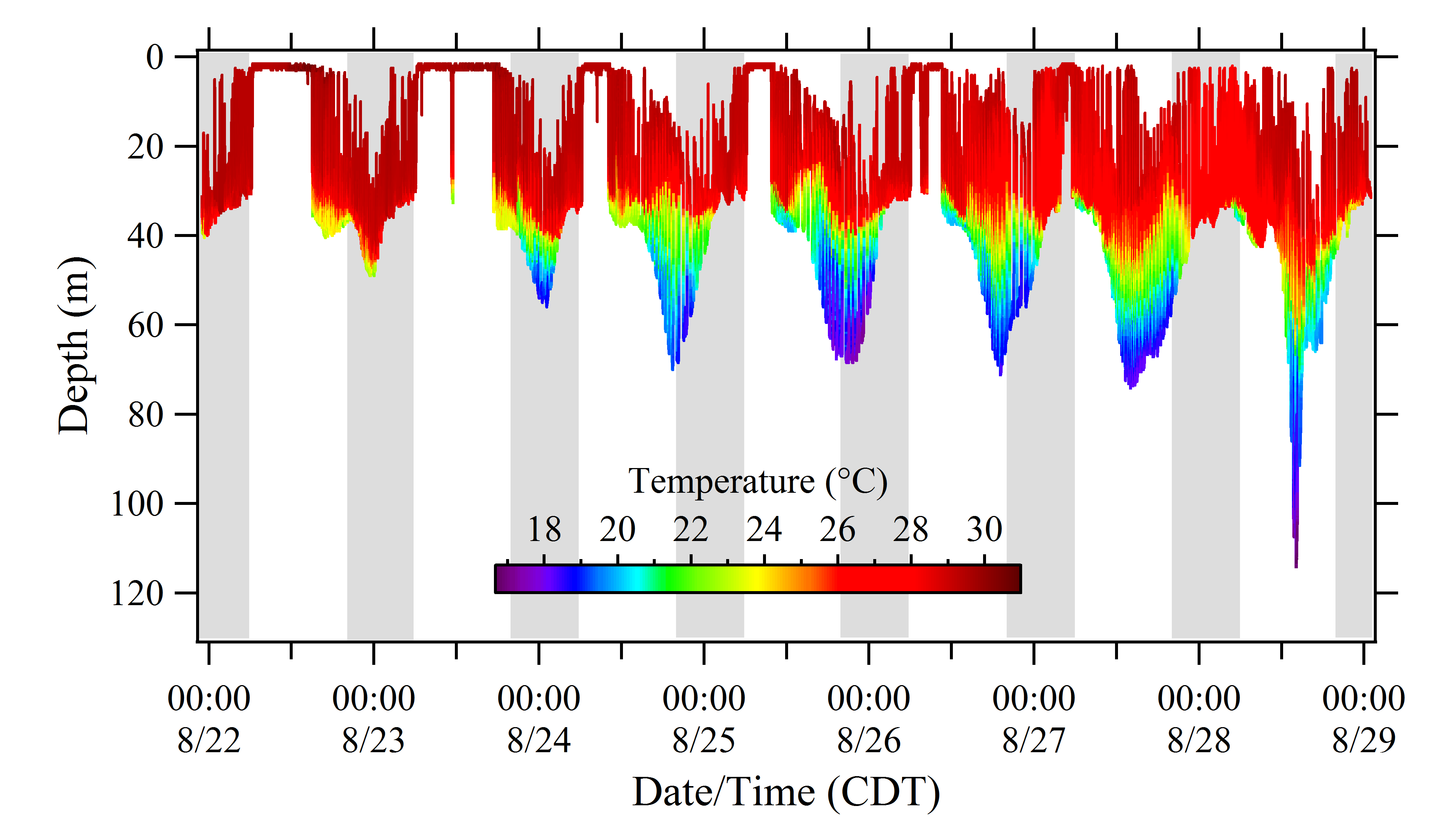

Supplement: S1 Fig — The reverse diel vertical migration transitions with a progressive decrease in daytime surface time and increase in vertical oscillations forming V-shaped profiles that span both day and night. (TIF) [file pone.0142156.s001.tif]
